# A Novel Gene *vp0610* Negatively Regulates Biofilm Formation in *Vibrio parahaemolyticus*

**DOI:** 10.3389/fmicb.2021.656380

**Published:** 2021-04-09

**Authors:** Fufeng Jiang, Tao Lei, Zhi Wang, Min He, Jumei Zhang, Juan Wang, Haiyan Zeng, Moutong Chen, Liang Xue, Qinghua Ye, Rui Pang, Shi Wu, Qihui Gu, Yu Ding, Qingping Wu

**Affiliations:** ^1^School of Food and Biological Engineering, Shaanxi University of Science and Technology, Xi’an, China; ^2^Guangdong Provincial Key Laboratory of Microbial Safety and Health, State Key Laboratory of Applied Microbiology Southern China, Institute of Microbiology, Guangdong Academy of Sciences, Guangzhou, China

**Keywords:** *vp0610*, biofilm formation, c-di-GMP, pull-down assay, *Vibrio parahaemolyticus*

## Abstract

*Vibrio parahaemolyticus* is an important foodborne pathogen and its biofilm formation ability facilitates its colonization and persistence in foods by protecting it from stresses including environmental variation and antibiotic exposure. Several important proteins are involved in biofilm formation; however, the identity and function of many remain unknown. In this study, we discovered a hypothetical protein, VP0610 that negatively regulates biofilm formation in *Vibrio parahaemolyticus*, and we found that the loss of *vp0610* typically results in pleiotropic phenotypes that contribute toward promoting biofilm formation, including significantly increased insoluble exopolysaccharide production and swimming motility, decreased soluble exopolysaccharide production, and decreased bis-(3′-5′)-cyclic dimeric guanosine monophosphate production. Pull-down assays revealed that VP0610 can interact with 180 proteins, some of which (Hfq, VP0710, VP0793, and CyaA) participate in biofilm formation. Moreover, deleting *vp0610* enhanced the expression of genes responsible for biofilm component (*flaE*), the sugar phosphotransferase system (PTS) EIIA component (*vp0710* and *vp0793*), and a high-density regulator of quorum sensing (*opaR*), while reducing the expression of the bis-(3′-5′)-cyclic dimeric guanosine monophosphate degradation protein (CdgC), resulting in faster biofilm formation. Taken together, our results indicate that *vp0610* is an integral member of the key biofilm regulatory network of *V. parahaemolyticus* that functions as a repressor of biofilm formation.

## Introduction

*Vibrio parahaemolyticus (V. parahaemolyticus)* is a versatile, opportunistic, Gram-negative, foodborne pathogen that represents a major global health threat as it causes infections in immunocompromised individuals and those with open wounds exposed to seawater (Daniels et al., 2000; O’Boyle and Boyd, 2014). In addition, *V. parahaemolyticus* disproportionately affects parts of the world that consume contaminated seafood (Baker-Austin et al., 2017). The main syndromes caused by *V. parahaemolyticus* include vomiting, diarrhea, gastroenteritis, wound infections, and septicemia (Lei et al., 2020). The ability of *V. parahaemolyticus* to cause infection is partially due to the virulence factors that it produces and the formation of persistent cells (Guo et al., 2019). *V. parahaemolyticus* can persist during infections and in seafood predominantly due to biofilm formation, which involves many specific structures and regulatory mechanisms, including flagella, type IV pili, extracellular polysaccharides (EPS), c-di-GMP, and quorum sensing (Yildiz and Visick, 2009). More than 90% of microorganisms can form biofilms, which are surface-attached microorganism communities enveloped in a matrix of EPS, proteins, and nucleic acids, which increases the infectivity and environmental survival of the pathogen, as well as cross-contamination (Flemming et al., 2016). The contribution of biofilms toward antimicrobial resistance and protection against host defenses has been well established in both chronic and acute infections (Gallego-Hernandez et al., 2020). Particularly, pathogenic bacteria have been shown to utilize elaborate regulatory mechanisms to modulate the expression of biofilm-associated traits.

In short, biofilm formation includes four stages: surface-adhesion, extracellular matrix production, biofilm maturation, and dispersal, which are each modulated by different structures and regulatory mechanisms (Teschler et al., 2015). Biofilm formation begins with attachment and settlement on biotic or abiotic surfaces (Mizan et al., 2016); indeed, it has been reported that motility mediated by polar flagella is crucial for initiating biofilm formation by increasing the chances of bacterial contact with a surface (Serra et al., 2013). Like flagellum, type IV pili are another type of extracellular organelles that are also crucial for the strength of bacterial adhesion on various surfaces (Maier and Wong, 2015). During each stage of biofilm formation, EPS acts as a “molecular glue” that allows bacteria to adhere to each other and surfaces, it provides protection against diverse stresses due to a changing environment, and a structure upon which to establish well-organized biofilm communities and nutrient gradients (Limoli et al., 2015), which plays an important part in biofilm maturation.

c-di-GMP is a central regulatory factor that participates in mannose sensitive hemagglutinin (MSHA) pilus formation, flagellum formation, biofilm matrix production and cell motility, thus governing surface attachment, biofilm formation and dispersal (Tolker-Nielsen, 2015; Zamorano-Sanchez et al., 2019). Cellular c-di-GMP interacts with different effector domains to confer bacteria with specific regulatory functions, such as adhesion and biofilm matrix synthesis; therefore, increased c-di-GMP levels can down-regulate flagellin expression to reduce or interfere with flagella motility (Navarro et al., 2011). Reversely, decreased c-di-GMP levels leads to the acceleration of cell motility in biofilm, thereby facilitating biofilm dispersal (Ha and O’Toole, 2015).

Multiple quorum-sensing (QS) systems exert distinct control over biofilm formation and dispersal in a manner that depends upon the accumulation or absence of autoinducers (Bridges and Bassler, 2019). When *V. parahaemolyticus* grows at a low cell density, the concentration of autoinducers in the extracellular environment is insufficient to bind membrane receptor kinases, which phosphorylate the phosphotransferase protein LuxU. In turn, LuxU phosphorylates the response regulator LuxO to activate the transcription of Qrr sRNAs, which promote the translation of the transcriptional regulator AphA and inhibit the translation of OpaR (Tu and Bassler, 2007; Tolker-Nielsen, 2015). In addition, AphA is required to produce the EPS matrix and biofilm formation. Although AphA activates biofilm formation, it directly inhibits the expression of MfpABC, a membrane fusion transporter that plays an important role in biofilm development (Ball et al., 2017). Together, biofilms represent a strongly protected growth mode that renders bacteria less sensitive to antibiotics and the host immune system (Del Pozo, 2018). Although pathogenic biofilms pose considerable challenges, such as persistent infection and bacterial retention on surfaces or in foods, to our lives, little is currently known about their development.

Recently, many studies have employed omics-based methods to screen potential genes involved in biofilm formation. Our previous comparative proteomics study of biofilm and planktonic-grown cells identified the biofilm-associated protein, *VP0610* (data not published); however, VP0610 was annotated as a hypothetical protein and the function of this protein in *V. parahaemolyticus* has not yet been identified. In this study, we found that *vp0610* can act on the PTS system and QS system to bring a series of phenotypic changes toward biofilm formation including swimming motility, EPS production and c-di-GMP. Understanding biofilm formation helps develop effective methods to control both food contamination and clinical infection caused by *V. parahaemolyticus*.

## Materials and Methods

### Bacterial Strains and Growth Conditions

*Vibrio parahaemolyticus* RIMD2210633 (kindly provided by Professor Jinquan Li, College of Food Science and Technology, Huazhong Agricultural University, China) and derivatives were cultured in Tryptic Soy Broth (TSB) supplemented with 3% NaCl (w/v) or on Chromogenic *Vibrio* Agar, purchased from the Guangdong Huankai Co., Ltd (Guangzhou, China) at 37°C. *Escherichia coli* strain SM17-λ-pir (kindly provided by Professor Hongyu Ou, School of Life Sciences and Biotechnology, Shanghai Jiao Tong University, China) was used for plasmid maintenance and conjugation, whereas *E. coli* strain BL21 (DE3) was used for protein expression. Both *E. coli* strains were routinely cultured in Lysogeny broth (LB) or on LB agar plates.

Antibiotics were used at the following concentrations: *E. coli*, ampicillin at 100 μg/mL, chloramphenicol at 34 μg/mL, kanamycin at 50 μg/mL; *V. parahaemolyticus*, chloramphenicol at 5 μg/mL. The media were supplemented with 0.5 mM IPTG to induce plasmid gene expression.

### Plasmid Construction

All primers used for strain and plasmid construction in this study are shown in Supplementary Table 1. The Δvp0610 null mutations and C-Δvp0610 complementary strains were made using long-flanking homology PCR, with 34 μg/mL chloramphenicol added for selection. For IPTG-inducible expression in *E. coli* BL21 (DE3), the *vp0610* coding sequence was amplified using an in-frame C-terminal 6× His tag from *V. parahaemolyticus* RIMD2210633 genomic DNA. Next, the PCR fragment was inserted into the multiple cloning site (MCS) of pET-B2M, which was derived from pET28a. Selection was conducted using 50 μg/mL kanamycin. The details of all strains and plasmids are listed in Table 1.

**TABLE 1:** Bacterial strains and plasmids used in this study.

**Strain or plasmid**	**Characteristic(s)**	**Source or reference**
**Strains**		
***V. parahaemolyticus***		
RIMD 2210633	Parent stain, serotype O3:K6	Zhang et al., 2012
Δ*vp0610* mutant	RIMD 2210633 Δ*vp0610*	This study
C-Δ*vp0610*	Insert *vp0610* to Δ*vp0610* mutant	This study
***E. coli***		
SM17 *λ-pir*	TpR SmR recA, thi, pro, hsdR-M+RP4: 2-Tc:Mu: Km Tn7 λpir	Zhang et al., 2012
BL21 (DE3)	F- ompT hsdSB (rB-, mB-) gal dcm endA1 lon- proUp::T7 RNAP::malQ-lacZ (TetS)	Götzke et al., 2014
**Plasmids**		
pDS132	R6K ori, *mobRP4 sacB* Cm^*r*^	Zhang et al., 2012
p_Δ_ *_*vp0610*_*	pDS132 with *vp0610* deletion	This study
pET B2M-vp0610	pET28a with vp0610	This study

### Construction of Deletion Mutants and Complementary Strains

A mutant *V. parahaemolyticus* strain was constructed by deleting the *vp0610* gene, as described previously with some minor modifications (Zhang et al., 2012). Briefly, competent SM17 *λ-pir* cells were transfected with the recombinant plasmid pDS132::*vp0610* and conjugated with wild-type (WT) strains. The exconjugants were selected on CHROM agar *Vibrio* media supplemented with 5 μg/mL chloramphenicol and 100 μg/mL ampicillin. Colonies with characteristics indicating a double homologous recombination event [resistance to 10% (w/v) sucrose, chloramphenicol sensitivity were isolated and target gene mutations were confirmed using PCR with specific primers (Supplementary Table 1)].

pDS132, containing the complete *vp0610* sequence with homology arm fragments, was propagated in *E. coli SM17-λ-pir* and conjugated to the corresponding *vp0610* mutant to generate a complementary strain using a similar process to those used for mutant construction. The presence of *vp0610* was verified using PCR and sequencing.

### Measurement of Biofilm Formation

Biofilms of the parental strain and isogenic *vp0610* mutant were produced as described previously, with minor modifications (O’Toole and Kolter, 1998). Briefly, each well of a 96-well polystyrene microtiter plate was inoculated with 200 μL of each culture diluted to OD_600_ = 0.2 with TSB and then incubated at 28°C without shaking for 24, 48, or 72 h. After planktonic cells had been removed, biofilms attached to the wall were washed with phosphate-buffered saline (PBS, pH 7.4), and stained with 230 μL of 0.1% (w/v) crystal violet (CV) solution shielded from light for 15 min. After floating color had been removed, biofilms were quantified by eluting CV with 230 μL acetic acid (33 %) and measuring absorbance at 590 nm (OD_590_) using a microplate spectrophotometer (EPOCH2, Bio Tek, Winooski, Vermont, United States). Biofilm formation ability was determined by calculating the biofilm formation index (BFI; Eq. 1), as follows:

(1) $$B\,F\,I=\frac{A\,B-C\,W}{G\,B-G\,W}$$

where AB represents the OD_590_ of CV-stained microorganisms, CW represents the OD_590_ of stained blank wells containing only TSB, GB represents the OD_600_ of the strain, and GW represents the OD_600_ of the blank well.

### Measurement of Growth Kinetics

To assess growth kinetics, the parental, mutant, and complementary strains were inoculated into a 96-well microplate (OD_600_ = 0.05) with a flat bottom and cover. Bacterial growth was monitored by measuring the optical density at 600 nm of each well every 30 min for 24 h at 30°C using a microplate spectrophotometer (EPOCH2) and data were recorded using Gen 5 (EPOCH2, Bio Tek, Winooski, Vermont, United States).

### Comparison of Strain Motility

Two types of motility occur depending on the environment: swimming in liquid media relies on the polar flagellum, whereas swarming on the surface of solid media depends upon lateral flagella (McCarter, 2004). We assessed the effect of mutations on swimming and swarming by examining bacterial motility on LB medium containing 0.2 and 1.5% (w/v) agar, respectively. Briefly, plates were spot inoculated with 2 μL of cell cultures (OD_600_ = 0.6) and incubated at 37°C for 8 or 48 h.

### EPS Analysis

Extracellular polysaccharides can be categorized into two main types: water-soluble EPS and water-insoluble EPS, both of which were prepared as described previously with minor modifications (Harimawan and Ting, 2016; Felz et al., 2019). Briefly, 2 mL of biofilm was collected from bacterial cultures grown for 24 h in 100 mL TSB and centrifuged at 5000 × *g* and 4°C for 20 min. The supernatant was used to measure water-soluble EPS, while the precipitate was re-suspended in 2 mL aqueous solution containing Buffer 1 (0.85% NaCl and 0.22% formaldehyde) at 80°C for 30 min to extract water-insoluble EPS by centrifugation at 4°C and 15000 × *g* for 30 min. Both soluble and insoluble EPS resuspensions (2 mL) were mixed with phenol (2 mL) and sulfuric acid (10 mL), placed in a boiling water bath for 15 min, and absorbance measured at 490 nm (OD_490_).

### RT-qPCR for Biofilm-Related Genes in *V. parahaemolyticus*

Wild-type, Δvp0610, and C-Δvp0610 strains were streaked on Chromogenic *Vibrio* Agar with 5 μg/mL chloramphenicol and sub-cultured in TSB for 24 h. Total RNA was extracted using RNAiso plus and treated using a PrimeScript^TM^ RT reagent kit with gDNA Eraser (Takara, Liaoning, China) according to the manufacturer’s instructions. qPCR was performed using a 2-Step RT-qPCR System (LightCycler^®^96, Roche, Basel, Switzerland) with TB Green^®^ Premix Ex Taq^TM^ (Tli RNaseH Plus). The primer sequences used for RT-qPCR are listed in Supplementary Table 1. Relative gene expression was calculated using the 2^–ΔΔ*t*^ method, with *16S rDNA* as a reference gene.

### Confocal Laser Scanning Microscope (CSLM) Analysis

Biofilms were cultured in 24-well polystyrene microtiter plates for 24 h and then 1 mL bacterial culture (OD_600_ = 0.20) was added to a cell culture coverslip. After cultivation, the biofilms on the coverslips were stained using a LIVE/DEAD^®^ BacLight Bacterial Viability Kit containing SYTO^®^ 9 green-fluorescent nucleic acid and propidium iodide red-fluorescent nucleic acid stain for 15 min in the dark and visualized using CSLM (LSM710, Zeiss, Jena, Germany). Biofilm images were processed using Zeiss Zen software (Zeiss).

### c-di-GMP Quantification Using Enzyme-Linked Immunosorbent Assay (ELISA)

c-di-GMP determination was performed as previously described with minor modifications (Meng et al., 2019). *V. parahaemolyticus* strains were grown for 12 and 24 h in TSB (3% NaCl) and centrifuged (2 mL) at 10000 × *g* for 10 min at 4°C. The cell pellet was washed three times and resuspended in 2 mL ice-cold PBS and sonicated in ice water for 15 min. After centrifugation, the supernatant containing the extracted c-di-GMP was collected and the pellet was resuspended in 2 mL of ice-cold PBS. This extraction step was repeated twice and intracellular c-di-GMP levels were determined using a c-di-GMP ELISA kit, which was purchased from Shanghai Ruifan Biotechnology Co., Ltd (Shanghai, China). Whole-cell protein concentration was determined using a BCA Protein Assay Kit, purchased from Beijing ComWin Biotech Co., Ltd (Beijing, China).

### Pull-Down Assay

*Escherichia coli* BL21 (DE3) transformed with a plasmid expressing VP0610 [pVP0610 (6× His)] was grown in LB with kanamycin (50 μg/mL) to an OD_600_ of 0.6. VP0610 expression was induced using 0.5 mM IPTG for 24 h at 20°C. The bacterial cultures were then centrifuged at 5000 × *g* for 10 min at 4°C, resuspended in binding buffer (20 mM sodium phosphate, 500 mM sodium chloride, 30 mM imidazole, pH 7.4), and treated with a high-pressure cell breaker. This process was repeated until the suspension became translucent and then the bacterial preparations were then filtered, immobilized using Ni Sepharose High Performance (GE Healthcare, Pittsburgh, Commonwealth of Pennsylvania, United States), and washed with 30% elution buffer. WT protein was loaded onto HisTrap Sepharose (GE Healthcare), eluted using different elution buffer concentrations, and analyzed using SDS-PAGE and silver staining. Next Western Blot was used to confirm the enrichment of VP0610 further. Protein bands were identified using mass spectrometry. Preliminary data were processed and converted into MGF format and used to retrieve data from the UniProt and NCBI databases. Data were analyzed using STRING (Damian et al., 2018).

### Statistical Analysis

Biological data for RT-qPCR experiments were 2^–ΔΔ*t*^ transformed before statistically analyzed. All assays were analyzed using one-way ANOVA with Dunnett’s multiple comparisons test. The growth curves, EPS production, RT-qPCR, and c-di-GMP experiments were analyzed using GraphPad Prism 6. Different letters for the biofilm assays, EPS production, c-di-GMP production and RT-qPCR indicate statistically significant differences in the individual strains for the various assays tested. Asterisks indicate *P*-value < 0.0001 (^****^); *P*-value < 0.001 (^∗∗∗^); *P*-value < 0.01 (^∗∗^); *P*-value < 0.05 (^∗^); and non-significant (ns).

## Results

### Bioinformatics Analysis

**FIGURE 1:**
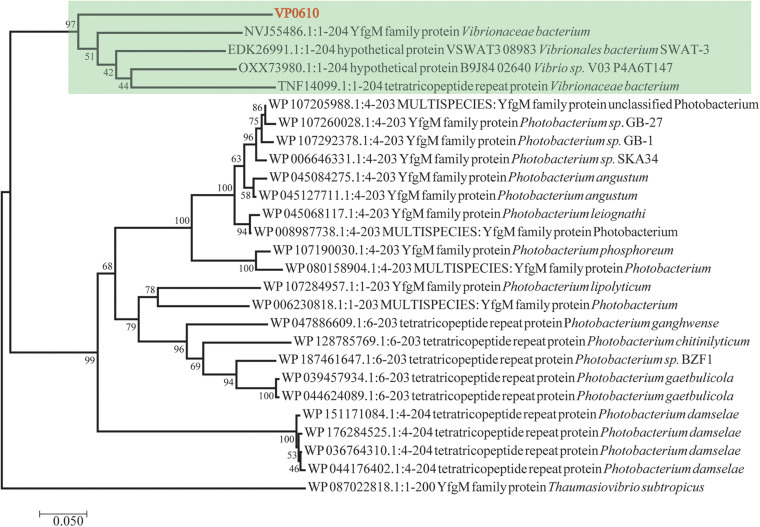
Evolutionary relationships of *vp0610*. The evolutionary history was inferred using the Neighbor-Joining method (Saitou and Nei, 1987). The optimal tree with the sum of branch length = 2.50135052 is shown. The percentage of replicate trees in which the associated taxa clustered together in the bootstrap test (1,000 replicates) are shown next to the branches (Felsenstein, 1985). The tree is drawn to scale, with branch lengths in the same units as those of the evolutionary distances used to infer the phylogenetic tree. The evolutionary distances were computed using the Poisson correction method (Zuckerkandl, 1965) and are in the units of the number of amino acid substitutions per site. The analysis involved 27 amino acid sequences. All positions containing gaps and missing data were eliminated. There was a total of 195 positions in the final dataset. Evolutionary analyses were conducted in MEGA7 (Sudhir et al., 2016).

Till now, *vp0610* is annotated as a hypothetical protein and we couldn’t find any research about VP0610 or its homologous protein in *Vibrio* spp. We applied Blast to search homologous protein and homology analysis revealed that VP0610 belongs to a YfgM family protein (Figure 1). However, some evidence suggest that YfgM serves as negative regulator of the transcriptional regulatory protein (RcsB)-dependent stress response pathway in *E. coli* (Westphal et al., 2012). RcsB is a transcriptional regulatory protein, controlling the expression of *lux* operon, thus affecting quorum sensing and biofilm formation (Gambino and Cappitelli, 2016), and the speculation is consistent with the comparative proteomic analysis (data not published). Furthermore, *vp0610* is probably related to biofilm formation.

### *vp0610* Delays Biofilm Formation

**FIGURE 2:**
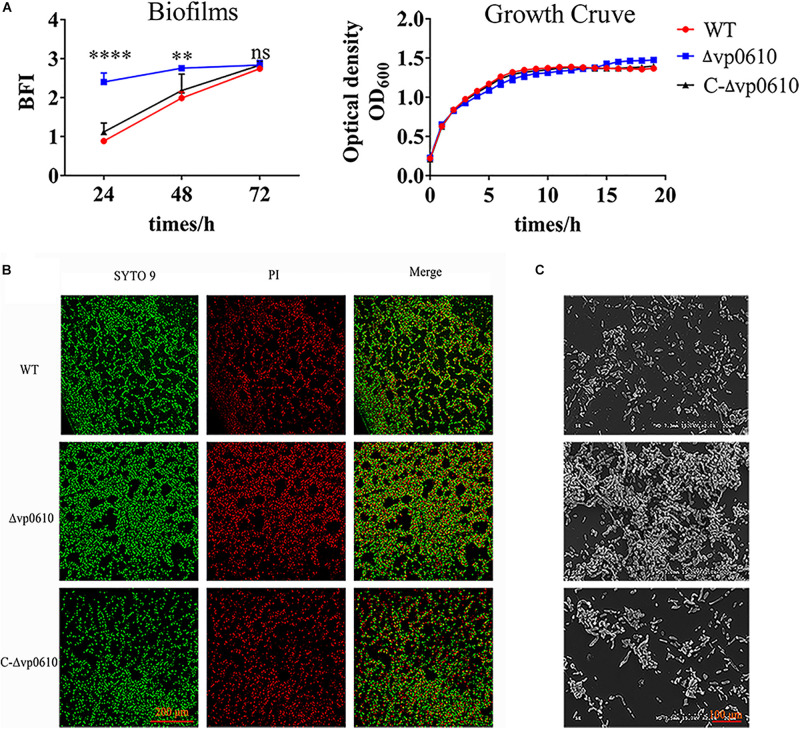
VP0610 inhibits biofilm formation in the initial stage. **(A)** Biofilm formation was determined at different times using a crystal violet staining method (Heo et al., 2019); growth curves were measured at 600 nm for 24 h. **(B)** Biofilm formation was visualized using confocal laser scanning microscopy. **(C)** Biofilm formation was visualized using scanning electron microscope. Significance was calculated by analysis of variance and Dunnett’s test comparison for differences from biofilm formation. **p < 0.01; ****p < 0.0001; ns, non-significant.

To elucidate the role of *vp0610* in *V. parahaemolyticus* biofilm formation, we monitored this process at 24, 48, and 72 h using CV staining. *vp0610* deletion significantly increased biofilm formation at 24 and 48 h, but increased biofilm formation by three-fold compared to the WT at 24 h. Interestingly, biofilm formation was faster in the WT than in the mutant after 24 h, meaning that this difference gradually decreased and disappeared completely by 72 h (Figure 2A). Moreover, quantitative analysis revealed that *vp0610* exerted a clear inhibitory effect during the early stages of biofilm formation. To exclude the bacterial growth influence, we monitored OD_600_ for 24 h and found that there was no significant difference between the WT and the mutant (Figure 2A). The results showed that *vp0610* delayed the biofilms formation at early stage, and then the difference gradually disappeared toward the end.

Next, direct microscopic visualization with a CLSM and SEM was further used to confirm the effect of *vp0610* on biofilm formation. The CLSM images showed that the mutant formed much thicker and more compact biofilms than the WT (Figure 2B), while the SEM images suggested that *vp0610* deletion dramatically increased bacterial surface density, consistent with the CV staining results (Figure 2C). Together, these findings suggest that Δvp0610 affects biofilm formation by altering cell adhesion and aggregation.

### *vp0610* Decreases Motility and Insoluble EPS Levels in *V. parahaemolyticus*

**FIGURE 3:**
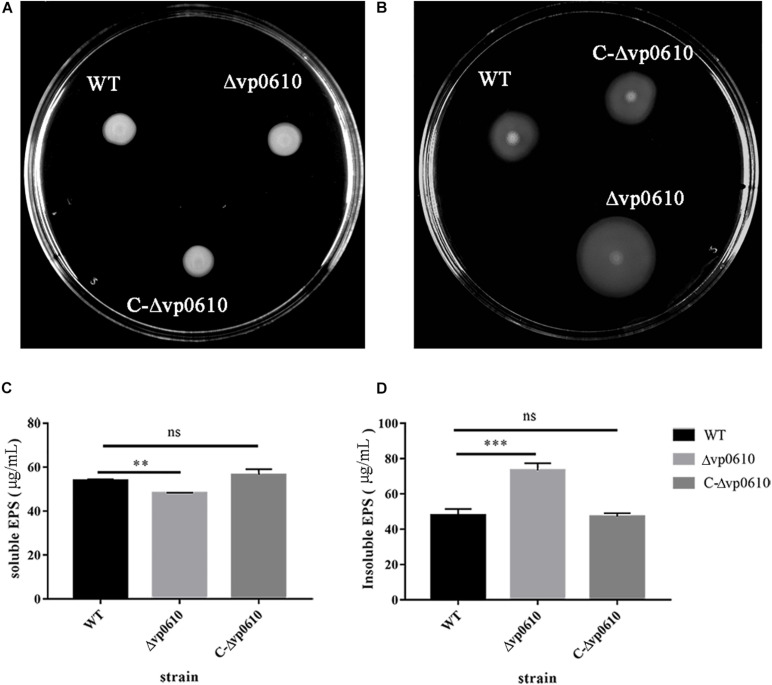
VP0610 can enhance swimming ability and insoluble EPS but decrease soluble EPS. **(A)** Three different strains of *V. parahaemolyticus* were inoculated into LB (0.2% agar) and incubated at 37°C for 8 h; **(B)** Three different strains of *V. parahaemolyticus* were inoculated into LB (1.5% agar) and incubated at 37°C for 48 h; **(C)** Soluble EPS determination; **(D)** Insoluble EPS determination. Soluble and insoluble EPS were extracted from biofilms attached to the bottom of beakers and then determined by phenol-vitriolic colorimetry. Significance was determined by analysis of variance and Dunnett’s test comparison for differences from EPS production. **p < 0.01; ***p < 0.001; ns, non-significant.

A previous study discovered that flagellar motility benefits initial colonization, adhesion, thereby promoting biofilm formation in both pathogenic and non-pathogenic *Vibrio* species (Khan et al., 2020). Therefore, we investigated whether *vp0610* affected *V. parahaemolyticus* biofilm formation by regulating its motility in semi-solid and on surfaces using swimming and swarming experiments, respectively. The WT strains grown in LB agar (0.2%) covered a much smaller diameter than the Δvp0610 strains (Figure 3A), indicating that *vp0610* negatively affects swimming; however, all strains displayed similar swarming abilities (Figure 3B), suggesting that *vp0610* does not affect swarming. Polar flagella mediate swimming motility. Our results suggested that *vp0610* had an inhibitory effect on the motility of polar flagella. Deleting *vp0610* resulted in the relieving of inhibition, causing the polar flagella to move quickly, and increasing the chances of cell contact with surfaces.

Extracellular polysaccharides can help the cells in a biofilm to adhere to the surface of the medium and form spatial structures, thereby protecting the cells in the biofilm (Limoli et al., 2015). To determine whether *vp0610* was required for EPS synthesis in *V. parahaemolyticus*, we measured the production of both water-soluble and -insoluble EPS in the WT and mutant strains. Interestingly, the mutant strain produced significantly less water-soluble EPS than the WT strain but produced considerably more water-insoluble EPS (Figures 3C,D). Thus, *vp0610* appears to positively regulate the production of soluble EPS and negatively regulate insoluble EPS production. The increase in water-insoluble EPS of the mutant enhances its adhesion and further facilitates biofilm formation, while the decrease of water-soluble EPS may result from the high density of bacteria in biofilm, which is consistent with the result of the biofilm measurement.

### VP0610 Reduces c-di-GMP Production

**FIGURE 4:**
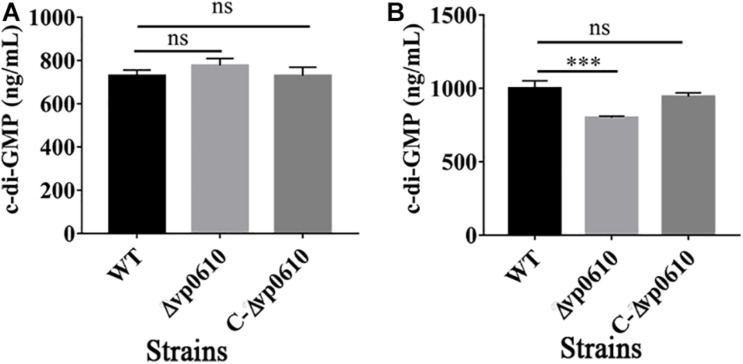
VP0610 modulates c-di-GMP production. The production of c-di-GMP in WT, Δvp0610 and C-Δvp0610 was quantified by measuring absorbance at 254 nm. **(A)** The production of c-di-GMP was measured at 12 h; **(B)** The production of c-di-GMP was measured at 24 h. Significance was measured by analysis of variance and Dunnett’s test comparison for differences from c-di-GMP production. ***p <0.001; ns, non-significant.

c-di-GMP can regulate the reversible transition of bacteria from a planktonic state into a biofilm state, with high c-di-GMP concentrations promoting biofilm formation and low concentrations promoting biofilm dispersion (Valentini and Filloux, 2016). Therefore, we examined whether *vp0610* affected c-di-GMP production by measuring the c-di-GMP levels at 12 and 24 h. Although the loss of *vp0610* did not alter c-di-GMP production at 12 h, a significant decrease was observed at 24 h (Figures 4A,B). Meanwhile, an obvious increase of in c-di-GMP production from 12 h to 24 h of WT was observed, but there was no change in the mutant strain. So, *vp0610* makes no difference at the early stage, but causes a significant decrease later, partly explaining why the biofilm of the mutant strain appeared to be slowly formed after 24 h.

### VP0610 Interacts With Other Proteins in *V. parahaemolyticus*

**FIGURE 5:**
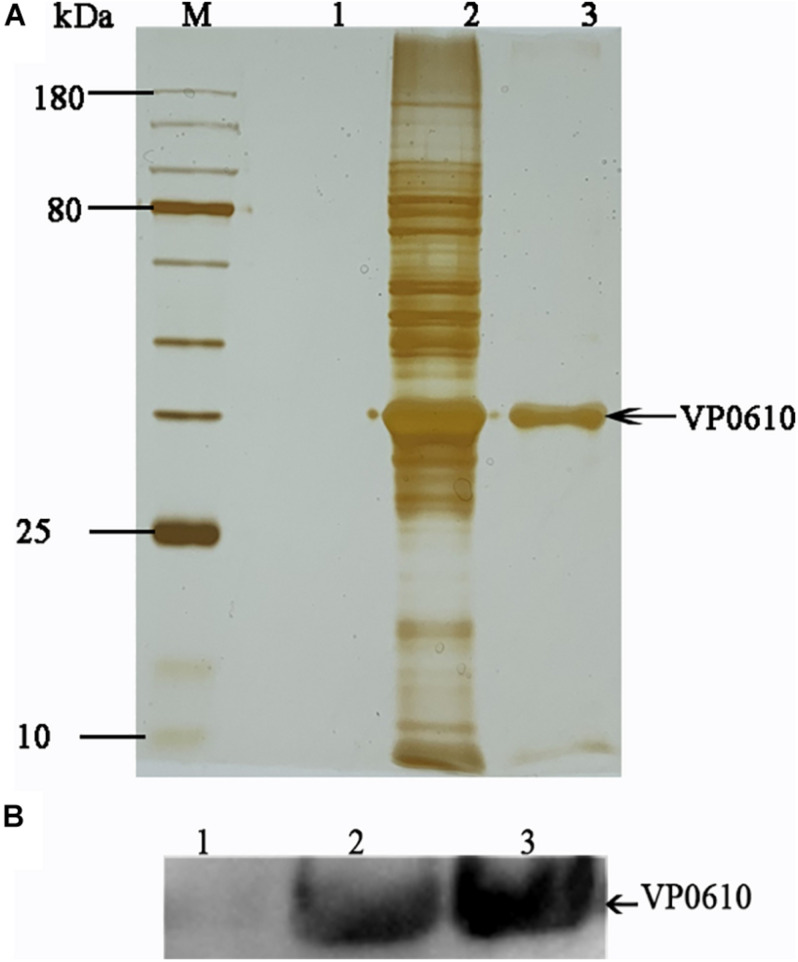
Pull down. **(A)** Pull down experiment was carried out to find proteins interacting with His-VP0610. Lane 1: WT protein was incubated with Ni Sepharose absence of His-VP0610; Lane 2: WT protein was incubated with Ni Sepharose present of His-VP0610; Lane 3: Purified His-VP0610. **(B)** Western blot with anti-VP0610. Lane 1: WT protein was incubated with Ni Sepharose absence of His-VP0610; Lane 2: Purified His-VP0610; Lane 3: WT protein was incubated with Ni Sepharose present of His-VP0610.

**FIGURE 6:**
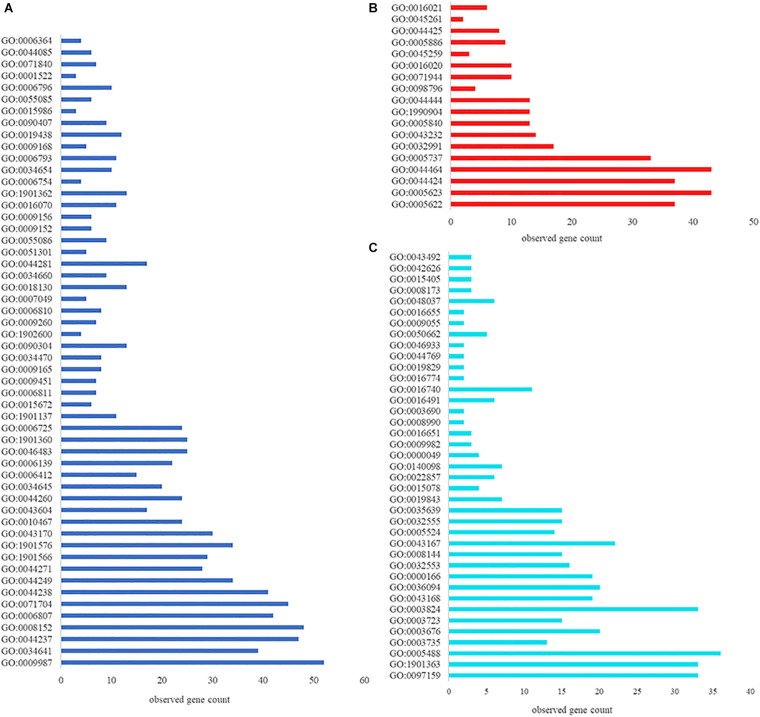
GO function annotation and enrichment analysis. **(A)** Biological process; **(B)** Cellular component; **(C)** Molecular function.

Having demonstrated that VP0610 participates in biofilm formation in *V. parahaemolyticus*, we aimed to identify new interaction partners of VP0610 by performing pull-down assays using hexahistidine-tagged VP0610 (His-VP0610) (Figure 5). Repeated experiments produced many protein bands that were mapped following in-gel tryptic digestion, revealing that 180 proteins interacted with His-VP0610 (Supplementary Table 2). Gene Ontology (GO) enrichment analysis indicated that these proteins could be divided into three groups: biological process, cellular component, and molecular function (Figure 6), while Kyoto Encyclopedia of Genes and Genomes (KEGG) pathway analysis revealed that the majority of the proteins participated in five main significant enrichment pathways, including RNA degradation, metabolic pathways, riboflavin metabolism, secondary metabolite biosynthesis, antibiotic biosynthesis, and lysine biosynthesis (Supplementary Table 3). Specifically, RNA binding protein (Hfq), adenylate cyclase (CyaA), and PTS-related protein components (VP0710 and VP0793) were identified as key proteins involved in biofilm formation via the quorum sensing and cAMP/CRP pathways (Supplementary Figure 1). Therefore, we will focus on cAMP/CRP pathways and quorum sensing to explore the effect of *vp0610* on biofilm formation.

### Effect of *vp0610* on Biofilm-Related Genes

**FIGURE 7:**
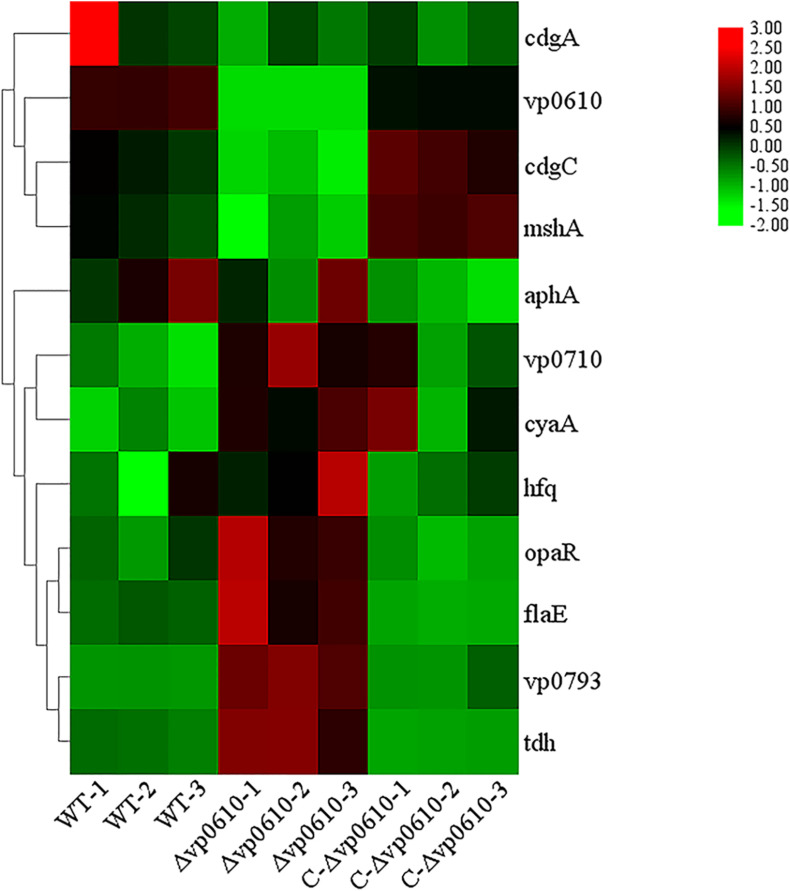
Biofilm-related gene expression analysis. Fold increase biofilm-related gene expression by RT-qPCR respect with WT, Δvp0610, and C-Δvp0610. This heat map was drawn by TB tools.

Based on the pull-down assay results, we subjected representative genes from the cAMP/CRP and quorum sensing pathways to RT-qPCR assays to determine how *vp0610* affected these genes. Importantly, the mutant strain displayed significantly higher levels of *vp0793, vp0710, opaR, flaE*, and *tdh* transcripts than the WT strain; however, *hfq, cyaA* and *aphA* expression were not significantly higher and *cdgC* and *mshA* expression were significantly lower (Figure 7). Our results showed *vp0610* could not only act on the cAMP/CRP pathways to affect biofilm formation, but also regulated flagellum-related genes through unknown pathways.

## Discussion

In this study, we found that the *vp0610* gene is related to biofilm formation and confirmed that *vp610* can significantly inhibit the initial stages (Figure 2A). Biofilm formation is a complex regulatory process that involves many factors; for instance, cell surface organelles, such as flagella and pili participate in the initial attachment to surfaces and can also aid migration along surfaces in some microorganisms, thereby promoting biofilm formation (Fong and Yildiz, 2015). Importantly, we found that Δvp0610 significantly decreased the swimming ability of *V. parahaemolyticus* (Figure 3A), which is mediated by polar flagella that control rapid movement, but not its swarming ability (Figure 3B), being controlled by lateral flagella that can make cells wriggle in situ. During the early stages of biofilm formation, cells will move rapidly to quickly sense the medium using flagella; however, *vp0610* deletion releases the inhibition of polar flagella, allowing bacteria to make rapid contact with the surface and form biofilms faster, consistent with our findings. Although the Δvp0610 strain formed biofilms more rapidly than the WT, the differences between the strains disappeared during the later stages (Figure 2A). Thus, it is important to understand the regulatory mechanisms underlying the role of VP0610 in flagellar motility in *V. parahaemolyticus* to determine how *vp0610* affects biofilms.

Extracellular polysaccharides is a critical component of the extracellular matrix that maintains biofilm architecture and protects bacteria from antimicrobials and host immune attack (Yu et al., 2015). In this study, we found that the mutant strains produced significantly less soluble EPS but significantly more insoluble EPS (Figures 3C,D). Previous studies have demonstrated that insoluble EPS acts as a molecular glue that helps bacterial cells to adhere to surfaces, thereby promoting irreversible colonization and accelerating biofilm formation. Soluble polysaccharides are often used as an emergency external energy reservoir for cells in biofilms, providing energy in unfavorable environments (Mattos-Graner et al., 2000; Limoli et al., 2015). Notably, we found that the biofilm formation rate of the mutant strain was significantly lower after 24 h of culturing, whereas biofilm formation in the WT strain continued to accelerate, ultimately yielding similar final amounts. This may be due to nutrient exhaustion in the medium after a long period of cultivation preventing the mutant strain from continuing to grow, yet the large store of soluble EPS in the WT strain continues to provide energy to maintain its growth. However, since the total energy in the culture medium is the same, the final level of biofilm formation is similar to that in the mutant strain once the extracellular soluble EPS has been consumed.

c-di-GMP is a secondary messenger that is found widely in both Gram-positive and Gram-negative bacteria and whose synthesis and degradation is controlled by diguanylate cyclase (DGCs) and phosphodiesterase (PDEs), respectively (Valentini and Filloux, 2016). c-di-GMP modulates biofilm determinants including flagella rotation, type IV pili retraction, EPS production, surface adhesin expression, antimicrobial resistance, other stress responses, secondary metabolite production, and biofilm dispersion (Romling et al., 2013). Quantitative analysis of c-di-GMP in this study suggested that *vp0610* does not affect c-di-GMP production at 12 h but significantly reduces production after 24 h compared to the WT (Figures 4A,B), which may explain the slow biofilm formation of Δvp0610 after this point.

**FIGURE 8:**
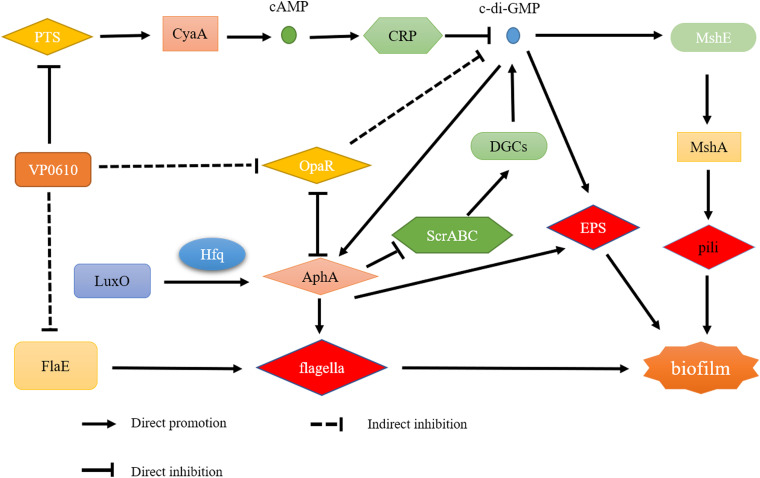
Regulatory network of VP0610.

As previously reported, there are four main regulatory pathways controlling biofilm in *Vibrio* spp. they include the VarS/VarA signaling system, the quorum sensing pathway, the cAMP/CRP signaling pathway, and the c-di-GMP signaling pathway. Our study revealed that VP0610 mainly acts through three major pathways, one of which is the cAMP/CRP signaling pathway. We found that VP0610 inhibits the expression of sugar PTS system EIIA components VP0710 and VP0793 on the cell membrane (Figure 7). cAMP-CRP in *E. coli* regulates curli fiber synthesis involved in adhesion and attachment; flagellum biosynthesis participated in motility, chemotaxis, and *rpoS* transcription, which governs many stress responses (Tsou et al., 2011; Liu et al., 2020). Here, we found that the presence of VP0610 leads to a decrease in the expression of sugar PTS system EIIA component (VP0710 and VP0793), then indirectly decreases the production of CyaA as well as cAMP, and further reducing the binding of cAMP to its receptor CRP. Further, we hypothesize that this process indirectly promotes c-di-GMP production (Figures 4A,B) and increases the expression of pilin MshA, which generally contributes to cell motility and promotes biofilm formation. The other pathway is the quorum sensing system. VP0610 can inhibit the transcription of *opaR*, while OpaR and AphA are the two main density-dependent core regulators of quorum sensing. HapR, a master regulator in *Vibrio cholerae*, negatively regulates biofilm formation by suppressing c-di-GMP production (Fong and Yildiz, 2008; Krasteva et al., 2010; Kalia et al., 2013; Biswas et al., 2020; Fernandez et al., 2020), consistent with our results; the c-di-GMP production of the mutant was significantly lower than that of the WT, resulting in a rapid biofilm formation in the WT after 24 h but a slow biofilm formation of the mutant. Therefore, we conclude that VP0610 has an indirect promoting effect on c-di-GMP production. Besides, AphA regulates guanylate cyclase (DGCs), responsible for producing c-di-GMP, by operon ScrABC. However, our result showed there was no significant difference between the WT and mutant. c-di-GMP as a core regulator of the transition between the biofilm lifestyle and the planktonic lifestyle of *V. parahaemolyticus* plays a decisive role in attachment and biofilm matrix production. Moreover, we found that VP0610 inhibits the production of insoluble polysaccharides and flagellin FlaE (Figure 8); however, the molecular details underlying this process remain unclear and should be investigated in future studies. We will apply multi-omics to study the detailed regulatory network.

## Conclusion

This study demonstrates VP0610 negatively regulates the early stages of *V. parahaemolyticus* biofilm formation by inhibiting the motility of polar flagella and insoluble EPS production. However, the biofilm formation difference between WT and mutant disappeared gradually. In addition, VP0610 acts on PTS system EIIA components VP0710 and VP0793 on the cell membrane to indirectly promote c-di-GMP production, causing a faster rate of biofilm formation. However, the specific regulatory pathways underlying this process remain unclear and should be investigated in future studies.

## Data Availability Statement

The original contributions presented in the study are included in the article/Supplementary Material, further inquiries can be directed to the corresponding author/s.

## Author Contributions

FJ, QW, ZW, and TL conceived and designed the experiments. FJ, TL, and MH performed the experiments. FJ, TL, JZ, JW, and ZW analyzed the data. FJ, HZ, MC, LX, QY, and TL drafted the manuscript. QW, RP, SW, YD, and QG supervised the project. All authors read and approved the final manuscript.

## Conflict of Interest

The authors declare that the research was conducted in the absence of any commercial or financial relationships that could be construed as a potential conflict of interest.
