# Supplementary material for: A Novel Gene vp0610 Negatively Regulates Biofilm Formation in Vibrio parahaemolyticus
Source: Front Microbiol. 2021 Apr 9;12:656380. doi: 10.3389/fmicb.2021.656380 (PMC8064395; doi:10.3389/fmicb.2021.656380)
Supplement: Supplementary file 1 [file Data_Sheet_1.docx]

**Supplementary materials**

**Table S1** Primers used in this study

| Primer | Sequence, 5’→3’ | Production/bp | Reference |
| --- | --- | --- | --- |
| *vp0610*-F_1_ *SacI* | CGAGCTCTCGCTACGACGGTCT | 524 | This study |
| *vp0610*-R_1_ | CCAATGCGGTCCTTATTCCTGTCCTCTTAA |  |  |
| *vp0610*-F_2_ | TTAAGAGGACAGGAATAAGGACCGCATTGG | 631 | This study |
| *vp0610*-R_2_ *SphI* | ACATGCATGCCGACCATTTGCCGTAC |  |  |
| sacB-F | CTTGGTAGCCATCTTCAGTT | 682 | This study |
| sacB-R | ATTACACGCCATGATATGCT |  |  |
| toxR-F | ATGCCATAGCATTTTTATCC | 368 | GENEWIZ, USA |
| toxR-R | CTGCGTTCTGATTTAATCTG |  |  |
| vp0610-F4 | TTAGGCATCGACAAGCACG | 1309 | This study |
| vp0610-R4 | GCTGACCACGCTCCACAAT |  | This study |
| q16SrRNA-F | TAAAATATGTAAGGGGTGAC |  | This study |
| q16SrRNA-R | GGATGTAACGCACTGAG |  |  |
| qtdh-F | TATCCTTGTTTGCCAGCGAG |  | (Ma et al., 2015) |
| qtdh-R | CTACGACGCACTTTTTGGG |  |  |
| qaphA-F | TGGCGGAATACCCAACAG |  | This study |
| qaphA-R | GTAACCCAGCGGCATTCA |  |  |
| qopaR-F | AGGGCATCGTTACCCAATC |  | This study |
| qopaR-R | TAAGTCAACATAGTCCGCATC |  |  |
| qvp0710-F | TGTTCTAAATGATACCGACAAAGC |  | This study |
| qvp0710-R | CAGCGTTCTTGGATTCGTCT |  |  |
| qmshA-F | CCGGCTGAGCTGCATTAC |  | This study |
| qmshA-R | CACCGTCGATAGAACTGTCTG |  |  |
| qvp0793-F | AAATCATCGCACCACTATCT |  | This study |
| qvp0793-R | CTGGCTTGATAGCAATACC |  |  |
| qcyaA-F | ATGGAAGAGTGTTACGACGAG |  | This study |
| qcyaA-R | CCACAAGTTCGAGCCAAAG |  |  |
| qflaE-F | ACAGTGCGGATAGCCAGTA |  | This study |
| qflaE-R | CTTTGAGTAGCGTCTCGTTT |  |  |
| qhfq-F | AACGGTATCAAACTACAAGGTC |  | This study |
| qhfq-R | CCGGCACAACTGTAGAAATCG |  |  |
| qcdgA-F | ATCGCACTTCCATCGTAA |  | This study |
| qcdgA-R | GCGGGTGTTATTTCTCAA |  |  |
| qcdgC-F | TATGTAAAGAGTCGGGTGAAG |  | This study |
| qcdgC-R | CCGCTTGGAGCAGATAA |  |  |
| qvp0610-F | TTGTGCTGGCGAGGAAG |  | This study |
| qvp0610-R | TGTCGTTTGCGTAGACTGG |  |  |

**Table S2** Interaction partners of VP0610

| prot_hit_num | prot_acc | prot_desc |
| --- | --- | --- |
| 1 | tr\|A0A0M3EB58\|A0A0M3EB58_VIBPH | (P)ppGpp synthetase OS=Vibrio parahaemolyticus OX=670 GN=AAY51_04925 PE=3 SV=1 |
| 2 | tr\|A0A0M3E5R0\|A0A0M3E5R0_VIBPH | 3,4-dihydroxy-2-butanone 4-phosphate synthase OS=Vibrio parahaemolyticus OX=670 GN=ribB PE=3 SV=1 |
| 3 | tr\|A0A072H799\|A0A072H799_VIBPH | 30S ribosomal protein S11 OS=Vibrio parahaemolyticus OX=670 GN=rpsK PE=3 SV=1 |
| 4 | tr\|A0A072IE55\|A0A072IE55_VIBPH | 30S ribosomal protein S13 OS=Vibrio parahaemolyticus OX=670 GN=rpsM PE=3 SV=1 |
| 5 | tr\|A0A072FYS6\|A0A072FYS6_VIBPH | 30S ribosomal protein S18 OS=Vibrio parahaemolyticus OX=670 GN=rpsR PE=3 SV=1 |
| 6 | tr\|A0A072GS55\|A0A072GS55_VIBPH | 30S ribosomal protein S19 OS=Vibrio parahaemolyticus OX=670 GN=rpsS PE=3 SV=1 |
| 7 | tr\|A0A0D1E8T7\|A0A0D1E8T7_VIBPH | 30S ribosomal protein S2 OS=Vibrio parahaemolyticus 49 OX=1288779 GN=rpsB PE=3 SV=1 |
| 8 | tr\|A0A0D1DWA1\|A0A0D1DWA1_VIBPH | 30S ribosomal protein S21 OS=Vibrio parahaemolyticus VP766 OX=1288782 GN=rpsU PE=3 SV=1 |
| 9 | tr\|A0A072JHR7\|A0A072JHR7_VIBPH | 30S ribosomal protein S5 OS=Vibrio parahaemolyticus OX=670 GN=rpsE PE=3 SV=1 |
| 10 | tr\|A0A072K176\|A0A072K176_VIBPH | 30S ribosomal protein S7 OS=Vibrio parahaemolyticus OX=670 GN=rpsG PE=3 SV=1 |
| 11 | tr\|A0A072G962\|A0A072G962_VIBPH | 30S ribosomal protein S8 OS=Vibrio parahaemolyticus OX=670 GN=rpsH PE=3 SV=1 |
| 12 | tr\|A0A0M3E0F2\|A0A0M3E0F2_VIBPH | 3-dehydroquinate synthase OS=Vibrio parahaemolyticus OX=670 GN=aroB PE=3 SV=1 |
| 13 | tr\|A0A0M3ECA5\|A0A0M3ECA5_VIBPH | 3-oxoacyl-[acyl-carrier-protein] synthase 3 OS=Vibrio parahaemolyticus OX=670 GN=fabH PE=3 SV=1 |
| 14 | tr\|A0A0D1ETC5\|A0A0D1ETC5_VIBPH | 50S ribosomal protein L13 OS=Vibrio parahaemolyticus 49 OX=1288779 GN=rplM PE=3 SV=1 |
| 15 | tr\|A0A072K649\|A0A072K649_VIBPH | 50S ribosomal protein L16 OS=Vibrio parahaemolyticus OX=670 GN=rplP PE=3 SV=1 |
| 16 | tr\|A0A072JZQ2\|A0A072JZQ2_VIBPH | 50S ribosomal protein L18 OS=Vibrio parahaemolyticus OX=670 GN=rplR PE=3 SV=1 |
| 17 | tr\|A0A0D1E320\|A0A0D1E320_VIBPH | 50S ribosomal protein L19 OS=Vibrio parahaemolyticus 49 OX=1288779 GN=rplS PE=3 SV=1 |
| 18 | sp\|P0A481\|RL20_VIBPA | 50S ribosomal protein L20 OS=Vibrio parahaemolyticus serotype O3:K6 (strain RIMD 2210633) OX=223926 GN=rplT PE=3 SV=1 |
| 19 | tr\|A0A072HFB5\|A0A072HFB5_VIBPH | 50S ribosomal protein L22 OS=Vibrio parahaemolyticus OX=670 GN=rplV PE=3 SV=1 |
| 20 | tr\|A0A072M095\|A0A072M095_VIBPH | 50S ribosomal protein L25 OS=Vibrio parahaemolyticus OX=670 GN=rplY PE=3 SV=1 |
| 21 | tr\|A0A072GSS4\|A0A072GSS4_VIBPH | 50S ribosomal protein L27 OS=Vibrio parahaemolyticus OX=670 GN=rpmA PE=3 SV=1 |
| 22 | tr\|A0A0M3E5U4\|A0A0M3E5U4_VIBPH | 50S ribosomal protein L29 OS=Vibrio parahaemolyticus OX=670 GN=rpmC PE=3 SV=1 |
| 23 | tr\|A0A072L946\|A0A072L946_VIBPH | 50S ribosomal protein L29 OS=Vibrio parahaemolyticus OX=670 GN=rpmC PE=3 SV=1 |
| 24 | tr\|A0A072GAC7\|A0A072GAC7_VIBPH | 50S ribosomal protein L3 OS=Vibrio parahaemolyticus OX=670 GN=rplC PE=3 SV=1 |
| 25 | tr\|A0A0M3ECX9\|A0A0M3ECX9_VIBPH | 50S ribosomal protein L32 OS=Vibrio parahaemolyticus OX=670 GN=rpmF PE=3 SV=1 |
| 26 | tr\|A0A072IEJ5\|A0A072IEJ5_VIBPH | 50S ribosomal protein L35 OS=Vibrio parahaemolyticus OX=670 GN=rpmI PE=3 SV=1 |
| 27 | tr\|A0A072I935\|A0A072I935_VIBPH | 50S ribosomal protein L6 OS=Vibrio parahaemolyticus OX=670 GN=rplF PE=3 SV=1 |
| 28 | tr\|A0A0D1E1A1\|A0A0D1E1A1_VIBPH | 50S ribosomal protein L7/L12 OS=Vibrio parahaemolyticus VP766 OX=1288782 GN=rplL PE=3 SV=1 |
| 29 | tr\|A0A072K9H8\|A0A072K9H8_VIBPH | 50S ribosomal protein L9 OS=Vibrio parahaemolyticus OX=670 GN=rplI PE=3 SV=1 |
| 30 | tr\|A0A0M3E2B1\|A0A0M3E2B1_VIBPH | 6,7-dimethyl-8-ribityllumazine synthase OS=Vibrio parahaemolyticus OX=670 GN=ribH PE=3 SV=1 |
| 31 | tr\|A0A0D1G353\|A0A0D1G353_VIBPH | 60 kDa chaperonin (Fragment) OS=Vibrio parahaemolyticus 49 OX=1288779 GN=groEL PE=3 SV=1 |
| 32 | tr\|A0A0D1U8B5\|A0A0D1U8B5_VIBPH | 60 kDa chaperonin OS=Vibrio parahaemolyticus OX=670 GN=groL PE=3 SV=1 |
| 33 | tr\|A0A0M3E2S5\|A0A0M3E2S5_VIBPH | ABC transporter ATP-binding protein OS=Vibrio parahaemolyticus OX=670 GN=AAY51_20520 PE=4 SV=1 |
| 34 | tr\|A0A072F7H7\|A0A072F7H7_VIBPH | Acetate kinase OS=Vibrio parahaemolyticus OX=670 GN=ackA PE=3 SV=1 |
| 35 | tr\|A0A2R9VQ07\|A0A2R9VQ07_VIBPH | Acetoin utilization protein AcuB OS=Vibrio parahaemolyticus OX=670 GN=BS585_21210 PE=4 SV=1 |
| 36 | tr\|A0A0D1CUP7\|A0A0D1CUP7_VIBPH | Acetylornithine aminotransferase OS=Vibrio parahaemolyticus VP766 OX=1288782 GN=argD PE=3 SV=1 |
| 37 | tr\|A0A0L8TID9\|A0A0L8TID9_VIBPH | Acetyltransferase component of pyruvate dehydrogenase complex OS=Vibrio parahaemolyticus OX=670 GN=aceF PE=3 SV=1 |
| 38 | tr\|A0A0D1GGA6\|A0A0D1GGA6_VIBPH | Acyl-[acyl-carrier-protein]--UDP-N-acetylglucosamine O-acyltransferase OS=Vibrio parahaemolyticus 901128 OX=1288792 GN=lpxA PE=3 SV=1 |
| 39 | tr\|A0A0D1E0H9\|A0A0D1E0H9_VIBPH | Alanine dehydrogenase OS=Vibrio parahaemolyticus VP766 OX=1288782 GN=H323_02465 PE=3 SV=1 |
| 40 | tr\|A0A072IK40\|A0A072IK40_VIBPH | Arginase OS=Vibrio parahaemolyticus OX=670 GN=BS585_08045 PE=4 SV=1 |
| 41 | tr\|A0A0M3ED14\|A0A0M3ED14_VIBPH | Arginine decarboxylase OS=Vibrio parahaemolyticus OX=670 GN=AAY51_03235 PE=4 SV=1 |
| 42 | tr\|A0A072IR40\|A0A072IR40_VIBPH | ATP synthase subunit b OS=Vibrio parahaemolyticus OX=670 GN=atpF PE=3 SV=1 |
| 43 | tr\|A0A072J6A3\|A0A072J6A3_VIBPH | ATP synthase subunit delta OS=Vibrio parahaemolyticus OX=670 GN=atpH PE=3 SV=1 |
| 44 | tr\|A0A072HIP8\|A0A072HIP8_VIBPH | ATP-dependent 6-phosphofructokinase OS=Vibrio parahaemolyticus OX=670 GN=pfkA PE=3 SV=1 |
| 45 | tr\|A0A0L8TZU9\|A0A0L8TZU9_VIBPH | ATP-dependent helicase HrpA OS=Vibrio parahaemolyticus OX=670 GN=WR32_02805 PE=4 SV=1 |
| 46 | tr\|A0A0M3E4A9\|A0A0M3E4A9_VIBPH | Bac_luciferase domain-containing protein OS=Vibrio parahaemolyticus OX=670 GN=AAY51_16710 PE=4 SV=1 |
| 47 | tr\|A0A0M3E800\|A0A0M3E800_VIBPH | Beta-methylgalactoside transporter permease OS=Vibrio parahaemolyticus OX=670 GN=mglC PE=3 SV=1 |
| 48 | tr\|A0A0M3E7C7\|A0A0M3E7C7_VIBPH | Carboxy-terminal protease OS=Vibrio parahaemolyticus OX=670 GN=AAY51_11920 PE=3 SV=1 |
| 49 | tr\|A0A0M3EC00\|A0A0M3EC00_VIBPH | Catalase OS=Vibrio parahaemolyticus OX=670 GN=katE PE=3 SV=1 |
| 50 | tr\|A0A0M3E5H1\|A0A0M3E5H1_VIBPH | Cell division protein FtsN OS=Vibrio parahaemolyticus OX=670 GN=AAY51_17760 PE=3 SV=1 |
| 51 | tr\|A0A0M3EA43\|A0A0M3EA43_VIBPH | Cell division protein FtsX OS=Vibrio parahaemolyticus OX=670 GN=ftsX PE=3 SV=1 |
| 52 | tr\|A0A072FFG4\|A0A072FFG4_VIBPH | Cell division protein FtsZ OS=Vibrio parahaemolyticus OX=670 GN=ftsZ PE=3 SV=1 |
| 53 | tr\|A0A0D1F211\|A0A0D1F211_VIBPH | Cell division protein ZapD OS=Vibrio parahaemolyticus 901128 OX=1288792 GN=zapD PE=3 SV=1 |
| 54 | tr\|A0A0D1EN21\|A0A0D1EN21_VIBPH | Cell division protein ZipA OS=Vibrio parahaemolyticus VP766 OX=1288782 GN=zipA PE=3 SV=1 |
| 55 | tr\|A0A072JVB5\|A0A072JVB5_VIBPH | Chaperone protein ClpB OS=Vibrio parahaemolyticus OX=670 GN=clpB PE=3 SV=1 |
| 56 | tr\|A0A072LLG8\|A0A072LLG8_VIBPH | Chromosomal replication initiator protein DnaA OS=Vibrio parahaemolyticus OX=670 GN=dnaA PE=3 SV=1 |
| 57 | tr\|A0A0M3E5R8\|A0A0M3E5R8_VIBPH | CinA-like protein OS=Vibrio parahaemolyticus OX=670 GN=AAY51_14590 PE=3 SV=1 |
| 58 | tr\|A0A0M3E6T4\|A0A0M3E6T4_VIBPH | Clp protease ClpP OS=Vibrio parahaemolyticus OX=670 GN=AAY51_17015 PE=4 SV=1 |
| 59 | tr\|A0A0D1DVS2\|A0A0D1DVS2_VIBPH | Cysteine desulfurase IscS OS=Vibrio parahaemolyticus VP766 OX=1288782 GN=iscS PE=3 SV=1 |
| 60 | tr\|A0A0D1F4K7\|A0A0D1F4K7_VIBPH | Cysteine desulfurase OS=Vibrio parahaemolyticus VP766 OX=1288782 GN=H323_03790 PE=3 SV=1 |
| 61 | tr\|A0A0M3E2T9\|A0A0M3E2T9_VIBPH | Cys-tRNA(Pro)/Cys-tRNA(Cys) deacylase OS=Vibrio parahaemolyticus OX=670 GN=AAY51_20605 PE=3 SV=1 |
| 62 | tr\|A0A0M3E2V5\|A0A0M3E2V5_VIBPH | DeoR faimly transcriptional regulator OS=Vibrio parahaemolyticus OX=670 GN=AAY51_17385 PE=4 SV=1 |
| 63 | tr\|A0A0M3E9U2\|A0A0M3E9U2_VIBPH | D-galactose transporter GalP OS=Vibrio parahaemolyticus OX=670 GN=AAY51_05545 PE=3 SV=1 |
| 64 | tr\|A0A0M3E8Q5\|A0A0M3E8Q5_VIBPH | Diaminopimelate decarboxylase OS=Vibrio parahaemolyticus OX=670 GN=lysA PE=3 SV=1 |
| 65 | tr\|A0A072F5U6\|A0A072F5U6_VIBPH | Dihydroorotate dehydrogenase (quinone) OS=Vibrio parahaemolyticus OX=670 GN=pyrD PE=3 SV=1 |
| 66 | tr\|A0A0M3E133\|A0A0M3E133_VIBPH | DNA helicase OS=Vibrio parahaemolyticus OX=670 GN=helD PE=3 SV=1 |
| 67 | tr\|A0A0M3E709\|A0A0M3E709_VIBPH | DNA-binding protein OS=Vibrio parahaemolyticus OX=670 GN=AAY51_15800 PE=3 SV=1 |
| 68 | tr\|A0A0D1EY94\|A0A0D1EY94_VIBPH | Elongation factor G OS=Vibrio parahaemolyticus VP766 OX=1288782 GN=fusA PE=3 SV=1 |
| 69 | tr\|A0A0M3EEJ6\|A0A0M3EEJ6_VIBPH | Endolytic murein transglycosylase OS=Vibrio parahaemolyticus OX=670 GN=mltG PE=3 SV=1 |
| 70 | tr\|A0A0D1DUM5\|A0A0D1DUM5_VIBPH | Esterase FrsA OS=Vibrio parahaemolyticus 49 OX=1288779 GN=frsA PE=3 SV=1 |
| 71 | tr\|A0A658H682\|A0A658H682_VIBPH | Flagellar L-ring protein FlgH OS=Vibrio parahaemolyticus OX=670 GN=DET53_1088 PE=3 SV=1 |
| 72 | tr\|A0A249W7T3\|A0A249W7T3_VIBPH | Formate acetyltransferase OS=Vibrio parahaemolyticus OX=670 GN=pflB PE=4 SV=1 |
| 73 | tr\|A0A0M3E729\|A0A0M3E729_VIBPH | Fumarate reductase flavoprotein subunit OS=Vibrio parahaemolyticus OX=670 GN=AAY51_07985 PE=3 SV=1 |
| 74 | tr\|A0A072IIM5\|A0A072IIM5_VIBPH | Fumarate reductase flavoprotein subunit OS=Vibrio parahaemolyticus OX=670 GN=frdA PE=3 SV=1 |
| 75 | tr\|A0A0D1DR21\|A0A0D1DR21_VIBPH | GlpT protein OS=Vibrio parahaemolyticus 49 OX=1288779 GN=glpT PE=4 SV=1 |
| 76 | tr\|A0A0M3ECR3\|A0A0M3ECR3_VIBPH | Glucosyltransferase I RfaG OS=Vibrio parahaemolyticus OX=670 GN=AAY51_04835 PE=4 SV=1 |
| 77 | tr\|A0A0D1EJL0\|A0A0D1EJL0_VIBPH | Glutamate 5-kinase OS=Vibrio parahaemolyticus 901128 OX=1288792 GN=proB PE=3 SV=1 |
| 78 | tr\|A0A0M3E329\|A0A0M3E329_VIBPH | Glycerol dehydrogenase OS=Vibrio parahaemolyticus OX=670 GN=gldA PE=4 SV=1 |
| 79 | tr\|A0A0L8SJC5\|A0A0L8SJC5_VIBPH | Glycerophosphodiester phosphodiesterase OS=Vibrio parahaemolyticus OX=670 GN=CA163_03770 PE=4 SV=1 |
| 80 | tr\|A0A0D1DM26\|A0A0D1DM26_VIBPH | Glycine--tRNA ligase beta subunit OS=Vibrio parahaemolyticus VP766 OX=1288782 GN=glyS PE=3 SV=1 |
| 81 | tr\|A0A072K8V0\|A0A072K8V0_VIBPH | Heat-shock protein HslJ OS=Vibrio parahaemolyticus OX=670 GN=C1S91_18200 PE=4 SV=1 |
| 82 | tr\|A0A0M3EC39\|A0A0M3EC39_VIBPH | HNHc domain-containing protein OS=Vibrio parahaemolyticus OX=670 GN=AAY51_01465 PE=4 SV=1 |
| 83 | tr\|A0A0M3E5M7\|A0A0M3E5M7_VIBPH | Hydrogenase 2 protein HybA OS=Vibrio parahaemolyticus OX=670 GN=AAY51_15945 PE=4 SV=1 |
| 84 | tr\|A0A0M3E450\|A0A0M3E450_VIBPH | Integration host factor subunit beta OS=Vibrio parahaemolyticus OX=670 GN=ihfB PE=3 SV=1 |
| 85 | tr\|A0A0M3E6L0\|A0A0M3E6L0_VIBPH | L-aspartate oxidase OS=Vibrio parahaemolyticus OX=670 GN=AAY51_11295 PE=3 SV=1 |
| 86 | tr\|A0A0D1EMB0\|A0A0D1EMB0_VIBPH | Lipoprotein OS=Vibrio parahaemolyticus 49 OX=1288779 GN=metQ PE=3 SV=1 |
| 87 | tr\|A0A0M3EAG0\|A0A0M3EAG0_VIBPH | L-lactate dehydrogenase OS=Vibrio parahaemolyticus OX=670 GN=lldD PE=3 SV=1 |
| 88 | tr\|A0A0M3E1L7\|A0A0M3E1L7_VIBPH | Macrodomain Ter protein OS=Vibrio parahaemolyticus OX=670 GN=matP PE=3 SV=1 |
| 89 | tr\|A0A0M3E7U1\|A0A0M3E7U1_VIBPH | Membrane protein OS=Vibrio parahaemolyticus OX=670 GN=AAY51_07925 PE=4 SV=1 |
| 90 | tr\|A0A0M3E0Q9\|A0A0M3E0Q9_VIBPH | Membrane protein OS=Vibrio parahaemolyticus OX=670 GN=AAY51_21675 PE=4 SV=1 |
| 91 | tr\|A0A072L5U8\|A0A072L5U8_VIBPH | Membrane protein OS=Vibrio parahaemolyticus OX=670 GN=BS585_06780 PE=4 SV=1 |
| 92 | tr\|A0A0L8U375\|A0A0L8U375_VIBPH | Membrane protein OS=Vibrio parahaemolyticus OX=670 GN=CA163_11830 PE=4 SV=1 |
| 93 | tr\|A0A658HGG5\|A0A658HGG5_VIBPH | Methyl-accepting chemotaxis protein OS=Vibrio parahaemolyticus OX=670 GN=DET53_101697 PE=4 SV=1 |
| 94 | tr\|A0A0D1E679\|A0A0D1E679_VIBPH | Methylglyoxal synthase OS=Vibrio parahaemolyticus 49 OX=1288779 GN=mgsA PE=3 SV=1 |
| 95 | tr\|A0A0L8S626\|A0A0L8S626_VIBPH | Molybdenum cofactor biosynthesis protein B OS=Vibrio parahaemolyticus OX=670 GN=moaB PE=3 SV=1 |
| 96 | tr\|A0A0L8SKN2\|A0A0L8SKN2_VIBPH | Molybdopterin molybdenumtransferase OS=Vibrio parahaemolyticus OX=670 GN=CGJ74_17960 PE=3 SV=1 |
| 97 | tr\|A0A0D1F3A2\|A0A0D1F3A2_VIBPH | Na(+)-translocating NADH-quinone reductase subunit A OS=Vibrio parahaemolyticus VP766 OX=1288782 GN=nqrA PE=3 SV=1 |
| 98 | tr\|A0A072IND5\|A0A072IND5_VIBPH | Na(+)-translocating NADH-quinone reductase subunit F OS=Vibrio parahaemolyticus OX=670 GN=nqrF PE=3 SV=1 |
| 99 | tr\|A0A0M3E382\|A0A0M3E382_VIBPH | N-acetylmuramoyl-l-alanine amidase I OS=Vibrio parahaemolyticus OX=670 GN=AAY51_22750 PE=4 SV=1 |
| 100 | tr\|A0A0D1EXT2\|A0A0D1EXT2_VIBPH | NAD kinase OS=Vibrio parahaemolyticus VP766 OX=1288782 GN=nadK PE=3 SV=1 |
| 101 | tr\|A0A227J159\|A0A227J159_VIBPH | NADH:ubiquinone reductase (Na(+)-transporting) subunit A (Fragment) OS=Vibrio parahaemolyticus OX=670 GN=CA163_31920 PE=4 SV=1 |
| 102 | tr\|A0A072JT35\|A0A072JT35_VIBPH | OmpA family protein OS=Vibrio parahaemolyticus OX=670 GN=C1S91_00325 PE=3 SV=1 |
| 103 | tr\|A0A0M3E760\|A0A0M3E760_VIBPH | Outer membrane protein assembly factor BamB OS=Vibrio parahaemolyticus OX=670 GN=bamB PE=3 SV=1 |
| 104 | tr\|A0A0M3E9C1\|A0A0M3E9C1_VIBPH | Peptidase B OS=Vibrio parahaemolyticus OX=670 GN=pepB PE=3 SV=1 |
| 105 | tr\|A0A0M3E8P1\|A0A0M3E8P1_VIBPH | Peptide ABC transporter ATP-binding protein OS=Vibrio parahaemolyticus OX=670 GN=AAY51_06850 PE=3 SV=1 |
| 106 | tr\|A0A0M3E5S4\|A0A0M3E5S4_VIBPH | Peptide deformylase OS=Vibrio parahaemolyticus OX=670 GN=def PE=3 SV=1 |
| 107 | tr\|A0A072F9P3\|A0A072F9P3_VIBPH | Peptidoglycan-associated protein OS=Vibrio parahaemolyticus OX=670 GN=pal PE=3 SV=1 |
| 108 | tr\|A0A072FQT7\|A0A072FQT7_VIBPH | Peptidyl-prolyl cis-trans isomerase OS=Vibrio parahaemolyticus OX=670 GN=BS585_00775 PE=3 SV=1 |
| 109 | tr\|A0A072F2W5\|A0A072F2W5_VIBPH | Peptidyl-prolyl cis-trans isomerase OS=Vibrio parahaemolyticus OX=670 GN=BS585_14080 PE=3 SV=1 |
| 110 | tr\|A0A072I5F9\|A0A072I5F9_VIBPH | Peptidylprolyl isomerase OS=Vibrio parahaemolyticus OX=670 GN=ppiD PE=4 SV=1 |
| 111 | tr\|A0A0F5TDH8\|A0A0F5TDH8_VIBPH | Periplasmic serine endoprotease DegP-like OS=Vibrio parahaemolyticus OX=670 GN=BS585_11985 PE=3 SV=1 |
| 112 | tr\|A0A072KT67\|A0A072KT67_VIBPH | Peroxidase OS=Vibrio parahaemolyticus OX=670 GN=BS585_21230 PE=4 SV=1 |
| 113 | tr\|A0A072JE78\|A0A072JE78_VIBPH | PhaC PHA synthase OS=Vibrio parahaemolyticus OX=670 GN=WR32_24045 PE=4 SV=1 |
| 114 | tr\|A0A0M3E9X4\|A0A0M3E9X4_VIBPH | Phosphatidylserine decarboxylase proenzyme OS=Vibrio parahaemolyticus OX=670 GN=psd PE=3 SV=1 |
| 115 | tr\|A0A0M3E431\|A0A0M3E431_VIBPH | Phosphoglucomutase OS=Vibrio parahaemolyticus OX=670 GN=AAY51_19665 PE=3 SV=1 |
| 116 | tr\|A0A0M3E0Y0\|A0A0M3E0Y0_VIBPH | Phosphoribosylglycinamide formyltransferase OS=Vibrio parahaemolyticus OX=670 GN=purN PE=3 SV=1 |
| 117 | tr\|A0A072JIB2\|A0A072JIB2_VIBPH | Prepilin peptidase OS=Vibrio parahaemolyticus OX=670 GN=C9I78_10590 PE=4 SV=1 |
| 118 | tr\|A0A0M3E3R6\|A0A0M3E3R6_VIBPH | Primosomal protein N~ OS=Vibrio parahaemolyticus OX=670 GN=priA PE=3 SV=1 |
| 119 | tr\|A0A0M3E8X2\|A0A0M3E8X2_VIBPH | Protease OS=Vibrio parahaemolyticus OX=670 GN=AAY51_07585 PE=4 SV=1 |
| 120 | tr\|A0A0D1GQZ4\|A0A0D1GQZ4_VIBPH | Protein adenylyltransferase SelO OS=Vibrio parahaemolyticus 901128 OX=1288792 GN=selO PE=3 SV=1 |
| 121 | tr\|A0A060IL13\|A0A060IL13_VIBPH | Protein RecA (Fragment) OS=Vibrio parahaemolyticus OX=670 GN=recA PE=3 SV=1 |
| 122 | tr\|A0A072IYW7\|A0A072IYW7_VIBPH | Pseudouridine synthase OS=Vibrio parahaemolyticus OX=670 GN=rluD PE=3 SV=1 |
| 123 | tr\|A0A072JPZ5\|A0A072JPZ5_VIBPH | Pseudouridine synthase OS=Vibrio parahaemolyticus OX=670 GN=rsuA PE=3 SV=1 |
| 124 | tr\|A0A0M3E809\|A0A0M3E809_VIBPH | PTS system trehalose(Maltose)-specific transporter subunits IIBC OS=Vibrio parahaemolyticus OX=670 GN=AAY51_08385 PE=4 SV=1 |
| 125 | tr\|A0A072LSJ8\|A0A072LSJ8_VIBPH | Pyruvate dehydrogenase E1 component OS=Vibrio parahaemolyticus OX=670 GN=aceE PE=4 SV=1 |
| 126 | tr\|A0A0M3EAZ6\|A0A0M3EAZ6_VIBPH | Replicative DNA helicase OS=Vibrio parahaemolyticus OX=670 GN=AAY51_03555 PE=3 SV=1 |
| 127 | tr\|A0A0M3EB21\|A0A0M3EB21_VIBPH | Rhodanese domain-containing protein OS=Vibrio parahaemolyticus OX=670 GN=AAY51_04740 PE=4 SV=1 |
| 128 | tr\|A0A0M3E4R3\|A0A0M3E4R3_VIBPH | Riboflavin biosynthesis protein RibD OS=Vibrio parahaemolyticus OX=670 GN=ribD PE=3 SV=1 |
| 129 | tr\|A0A0L8S3Z5\|A0A0L8S3Z5_VIBPH | Ribonuclease E OS=Vibrio parahaemolyticus OX=670 GN=rne PE=3 SV=1 |
| 130 | tr\|A0A0F5SKN9\|A0A0F5SKN9_VIBPH | Ribonuclease R OS=Vibrio parahaemolyticus OX=670 GN=rnr PE=3 SV=1 |
| 131 | tr\|A0A0M3E1A5\|A0A0M3E1A5_VIBPH | Ribosomal protein S12 methylthiotransferase RimO OS=Vibrio parahaemolyticus OX=670 GN=rimO PE=3 SV=1 |
| 132 | tr\|A0A0D1E8Q7\|A0A0D1E8Q7_VIBPH | Ribosomal RNA large subunit methyltransferase G OS=Vibrio parahaemolyticus 49 OX=1288779 GN=rlmG PE=3 SV=1 |
| 133 | tr\|A0A0M3E6L8\|A0A0M3E6L8_VIBPH | Ribosomal RNA small subunit methyltransferase I OS=Vibrio parahaemolyticus OX=670 GN=rsmI PE=3 SV=1 |
| 134 | tr\|A0A0M3E9S3\|A0A0M3E9S3_VIBPH | RNA polymerase-binding transcription factor DksA OS=Vibrio parahaemolyticus OX=670 GN=dksA PE=3 SV=1 |
| 135 | tr\|A0A072G9G3\|A0A072G9G3_VIBPH | RNA-binding protein Hfq OS=Vibrio parahaemolyticus OX=670 GN=hfq PE=3 SV=1 |
| 136 | tr\|A0A0M3E8H0\|A0A0M3E8H0_VIBPH | Selenate reductase subunit YgfK OS=Vibrio parahaemolyticus OX=670 GN=AAY51_09365 PE=4 SV=1 |
| 137 | tr\|A0A0M3E676\|A0A0M3E676_VIBPH | Soluble pyridine nucleotide transhydrogenase OS=Vibrio parahaemolyticus OX=670 GN=sthA PE=3 SV=1 |
| 138 | tr\|A0A072JBM7\|A0A072JBM7_VIBPH | Spermidine N1-acetyltransferase OS=Vibrio parahaemolyticus OX=670 GN=speG PE=4 SV=1 |
| 139 | tr\|A0A0M3E1T6\|A0A0M3E1T6_VIBPH | Stationary phase/starvation inducible regulatory protein CspD OS=Vibrio parahaemolyticus OX=670 GN=AAY51_18650 PE=4 SV=1 |
| 140 | tr\|A0A0M3E9X0\|A0A0M3E9X0_VIBPH | Succinate dehydrogenase iron-sulfur subunit OS=Vibrio parahaemolyticus OX=670 GN=AAY51_07980 PE=3 SV=1 |
| 141 | tr\|A0A0M3E500\|A0A0M3E500_VIBPH | Succinate dehydrogenase OS=Vibrio parahaemolyticus OX=670 GN=sdhC PE=4 SV=1 |
| 142 | tr\|A0A0D1F5M3\|A0A0D1F5M3_VIBPH | Threonine--tRNA ligase OS=Vibrio parahaemolyticus 901128 OX=1288792 GN=thrS PE=3 SV=1 |
| 143 | tr\|A0A0M3E289\|A0A0M3E289_VIBPH | Tol-Pal system protein TolR OS=Vibrio parahaemolyticus OX=670 GN=tolR PE=3 SV=1 |
| 144 | tr\|A0A0M3E1B0\|A0A0M3E1B0_VIBPH | Toprim domain-containing protein OS=Vibrio parahaemolyticus OX=670 GN=AAY51_18670 PE=4 SV=1 |
| 145 | tr\|A0A0M3EC18\|A0A0M3EC18_VIBPH | Transcriptional regulator OS=Vibrio parahaemolyticus OX=670 GN=AAY51_01290 PE=4 SV=1 |
| 146 | tr\|A0A0M3E869\|A0A0M3E869_VIBPH | Transcriptional regulator OS=Vibrio parahaemolyticus OX=670 GN=AAY51_09820 PE=3 SV=1 |
| 147 | tr\|A0A0M3E5K0\|A0A0M3E5K0_VIBPH | Transcriptional regulator OS=Vibrio parahaemolyticus OX=670 GN=AAY51_14140 PE=3 SV=1 |
| 148 | tr\|A0A0M3E6K5\|A0A0M3E6K5_VIBPH | Transcriptional regulator OS=Vibrio parahaemolyticus OX=670 GN=AAY51_14765 PE=3 SV=1 |
| 149 | tr\|A0A0M3E5P7\|A0A0M3E5P7_VIBPH | Transcriptional regulator OS=Vibrio parahaemolyticus OX=670 GN=AAY51_16060 PE=4 SV=1 |
| 150 | tr\|A0A0M3EA37\|A0A0M3EA37_VIBPH | Trehalose repressor OS=Vibrio parahaemolyticus OX=670 GN=treR PE=4 SV=1 |
| 151 | tr\|A0A072JSV4\|A0A072JSV4_VIBPH | Trigger factor OS=Vibrio parahaemolyticus OX=670 GN=tig PE=3 SV=1 |
| 152 | tr\|A0A0D1EP51\|A0A0D1EP51_VIBPH | tRNA (guanine-N(7)-)-methyltransferase OS=Vibrio parahaemolyticus 49 OX=1288779 GN=trmB PE=3 SV=1 |
| 153 | tr\|A0A072KML1\|A0A072KML1_VIBPH | tRNA pseudouridine synthase A OS=Vibrio parahaemolyticus OX=670 GN=truA PE=3 SV=1 |
| 154 | tr\|A0A072IDS0\|A0A072IDS0_VIBPH | Tryptophan synthase beta chain OS=Vibrio parahaemolyticus OX=670 GN=trpB PE=3 SV=1 |
| 155 | tr\|A0A0M3E7Q0\|A0A0M3E7Q0_VIBPH | Tyrosine recombinase XerD OS=Vibrio parahaemolyticus OX=670 GN=xerD PE=3 SV=1 |
| 156 | tr\|A0A0M3E5C7\|A0A0M3E5C7_VIBPH | Ubiquinol oxidase subunit 2 OS=Vibrio parahaemolyticus OX=670 GN=AAY51_20865 PE=3 SV=1 |
| 157 | tr\|A0A0D1E4I6\|A0A0D1E4I6_VIBPH | UDP-glucose 6-dehydrogenase OS=Vibrio parahaemolyticus VP766 OX=1288782 GN=H323_13825 PE=3 SV=1 |
| 158 | tr\|A0A0M3EBT5\|A0A0M3EBT5_VIBPH | UDP-N-acetylmuramoyl-L-alanyl-D-glutamate--2,6-diaminopimelate ligase OS=Vibrio parahaemolyticus OX=670 GN=murE PE=3 SV=1 |
| 159 | tr\|A0A4Z7AEJ7\|A0A4Z7AEJ7_VIBPH | Uncharacterized protein (Fragment) OS=Vibrio parahaemolyticus OX=670 GN=CGH73_26505 PE=4 SV=1 |
| 160 | tr\|A0A0M3E902\|A0A0M3E902_VIBPH | Uncharacterized protein OS=Vibrio parahaemolyticus OX=670 GN=AAY51_07185 PE=4 SV=1 |
| 161 | tr\|A0A0M3E5V7\|A0A0M3E5V7_VIBPH | Uncharacterized protein OS=Vibrio parahaemolyticus OX=670 GN=AAY51_16410 PE=4 SV=1 |
| 162 | tr\|A0A0M3E4A6\|A0A0M3E4A6_VIBPH | Uncharacterized protein OS=Vibrio parahaemolyticus OX=670 GN=AAY51_18790 PE=4 SV=1 |
| 163 | tr\|A0A660AQ38\|A0A660AQ38_VIBPH | Uncharacterized protein OS=Vibrio parahaemolyticus OX=670 GN=CGI34_23030 PE=4 SV=1 |
| 164 | tr\|A0A658H5G8\|A0A658H5G8_VIBPH | Uncharacterized protein OS=Vibrio parahaemolyticus OX=670 GN=DET53_11016 PE=4 SV=1 |
| 165 | tr\|A0A0M3EAK2\|A0A0M3EAK2_VIBPH | UPF0267 protein AAY51_09270 OS=Vibrio parahaemolyticus OX=670 GN=AAY51_09270 PE=3 SV=1 |
| 166 | tr\|A0A0D1CY39\|A0A0D1CY39_VIBPH | UPF0283 membrane protein H323_09255 OS=Vibrio parahaemolyticus VP766 OX=1288782 GN=H323_09255 PE=3 SV=1 |
| 167 | tr\|A0A0M3E5H6\|A0A0M3E5H6_VIBPH | UTP--glucose-1-phosphate uridylyltransferase OS=Vibrio parahaemolyticus OX=670 GN=AAY51_13710 PE=4 SV=1 |
| 168 | tr\|A0A0D1F6J7\|A0A0D1F6J7_VIBPH | Whole genome shotgun sequence (Fragment) OS=Vibrio parahaemolyticus VP766 OX=1288782 GN=H323_03830 PE=3 SV=1 |
| 169 | tr\|A0A0D1EFN6\|A0A0D1EFN6_VIBPH | Whole genome shotgun sequence OS=Vibrio parahaemolyticus 49 OX=1288779 GN=H320_03890 PE=4 SV=1 |
| 170 | tr\|A0A0D1DUM7\|A0A0D1DUM7_VIBPH | Whole genome shotgun sequence OS=Vibrio parahaemolyticus 49 OX=1288779 GN=H320_05740 PE=4 SV=1 |
| 171 | tr\|A0A0D1FJV9\|A0A0D1FJV9_VIBPH | Whole genome shotgun sequence OS=Vibrio parahaemolyticus 49 OX=1288779 GN=H320_11650 PE=4 SV=1 |
| 172 | tr\|A0A0D1E8F0\|A0A0D1E8F0_VIBPH | Whole genome shotgun sequence OS=Vibrio parahaemolyticus 49 OX=1288779 GN=H320_13575 PE=4 SV=1 |
| 173 | tr\|A0A0D1G1Y6\|A0A0D1G1Y6_VIBPH | Whole genome shotgun sequence OS=Vibrio parahaemolyticus 49 OX=1288779 GN=H320_15610 PE=3 SV=1 |
| 174 | tr\|A0A0D1E4D6\|A0A0D1E4D6_VIBPH | Whole genome shotgun sequence OS=Vibrio parahaemolyticus 49 OX=1288779 GN=H320_18420 PE=3 SV=1 |
| 175 | tr\|A0A0D1GIH2\|A0A0D1GIH2_VIBPH | Whole genome shotgun sequence OS=Vibrio parahaemolyticus 901128 OX=1288792 GN=H334_11850 PE=3 SV=1 |
| 176 | tr\|A0A0D1ENE4\|A0A0D1ENE4_VIBPH | Whole genome shotgun sequence OS=Vibrio parahaemolyticus 901128 OX=1288792 GN=H334_15120 PE=4 SV=1 |
| 177 | tr\|A0A0D1DGJ7\|A0A0D1DGJ7_VIBPH | Whole genome shotgun sequence OS=Vibrio parahaemolyticus VP766 OX=1288782 GN=H323_02305 PE=3 SV=1 |
| 178 | tr\|A0A0D1DKU2\|A0A0D1DKU2_VIBPH | Whole genome shotgun sequence OS=Vibrio parahaemolyticus VP766 OX=1288782 GN=H323_07520 PE=4 SV=1 |
| 179 | tr\|A0A0D1FA36\|A0A0D1FA36_VIBPH | Whole genome shotgun sequence OS=Vibrio parahaemolyticus VP766 OX=1288782 GN=H323_21530 PE=4 SV=1 |
| 180 | tr\|A0A072IGX2\|A0A072IGX2_VIBPH | YjbQ family protein OS=Vibrio parahaemolyticus OX=670 GN=BS585_03965 PE=4 SV=1 |

**Table S3** KEGG pathway analysis

| **term ID** | **term description** | **observed gene count** | | **background gene count** | **false discovery rate** | **matching proteins in your network (labels)** |
| --- | --- | --- | --- | --- | --- | --- |
| vpa03010 | Ribosome | | 24 | 55 | 7.53E-23 | *rplC,rplF,rplI,rplL,rplM,rplP,rplR,rplS,rplT,rplV,rplY,rpmA,*  *rpmC,rpmF,rpmI,rpsB,rpsE,rpsG,rpsH,rpsK,rpsM,rpsR,rpsS,rpsU* |
| vpa03018 | RNA degradation | | 5 | 21 | 0.0018 | *vp1890,groL1,hfq,pfkA,rne* |
| vpa01100 | Metabolic pathways | | 25 | 663 | 0.0037 | *vp0363,vp0679,vp0681,vp0843,vp2518,vp2519,argD,aroB,atpF,atpH,fabH1,*  *iscS,lldD,lpxA,lysA,nadK,pepB,pfkA,proB,psd,purN,pyrD,ribH,sthA,trpB1* |
| vpa00740 | Riboflavin metabolism | | 3 | 12 | 0.0245 | *vp0679,vp0681,ribH* |
| vpa01110 | Biosynthesis of secondary metabolites | | 13 | 292 | 0.0245 | *vp0679,vp0681,vp0843,vp2518,vp2519,argD,aroB,lysA,*  *pfkA,psd,purN,ribH,trpB1* |
| vpa01130 | Biosynthesis of antibiotics | | 10 | 201 | 0.0296 | *vp0843,vp2518,vp2519,argD,aroB,lysA,pfkA,proB,purN,trpB1* |
| vpa00300 | Lysine biosynthesis | | 3 | 17 | 0.0329 | *argD,lysA,murE* |


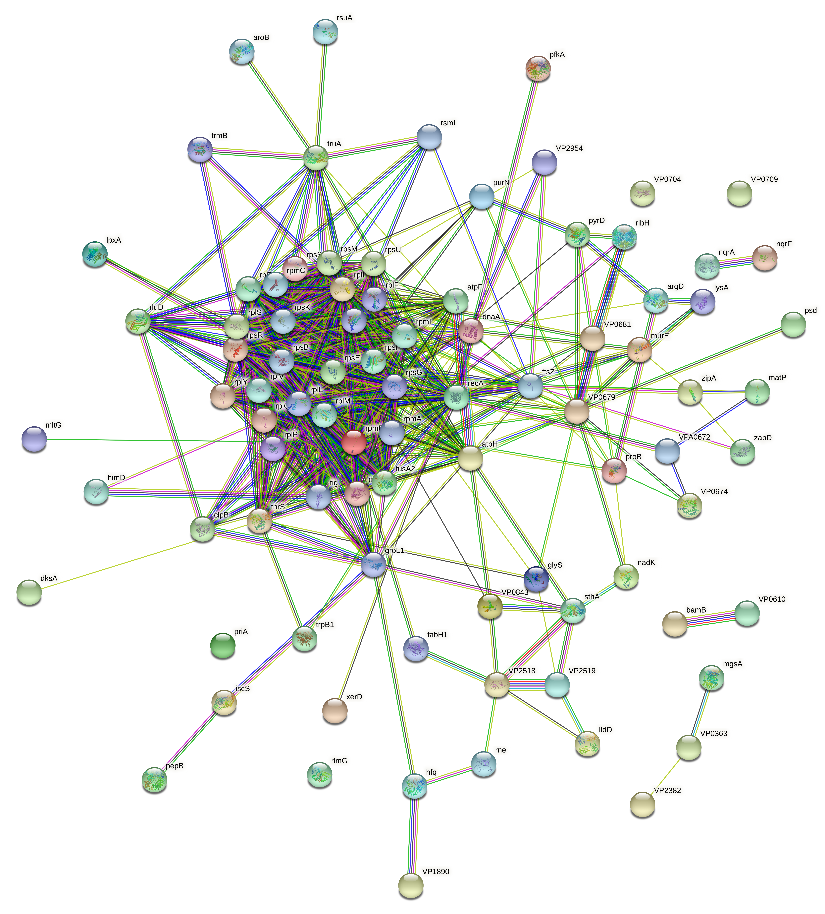


**Figure S1. Interaction analysis of partner proteins.** Network nodes represent proteins, splice isoforms or post-translational modifications are collapsed, i.e. each node represents all the proteins produced by a single, protein-coding gene locus. Edges represent protein-protein associations, associations are meant to be specific and meaningful, i.e. proteins jointly contribute to a shared function; this does not necessarily mean they are physically binding each other.

Ma, Y.-J., Sun, X.-H., Xu, X.-Y., Zhao, Y., Pan, Y.-J., Hwang, C.-A., et al. (2015). Investigation of Reference Genes in Vibrio parahaemolyticus for Gene Expression Analysis Using Quantitative RT-PCR. *PloS one* 10(12)**,** e0144362-e0144362. doi: 10.1371/journal.pone.0144362.
